# When age meets genetics: refining heart failure risk prediction in young atrial fibrillation

**DOI:** 10.1093/europace/euaf201

**Published:** 2025-09-01

**Authors:** Bin Deng, Wenhua Liu

**Affiliations:** Department of Cardiovascular Medicine, Shenzhen Bao’an Chinese Medicine Hospital, No.25 Yu'an 2nd Road, xin'an Subdistrict, Ban'an District, Shenzhen, 518100, China; Department of Endocrinology, Shenzhen Bao’an Authentic TCM Therapy Hospital, No.99 Lai'an Road, xixiang Subdistrict, Bao'an District, Shenzhen 518102, China

We read with interest Ahn *et al*.’s analysis from the UK Biobank evaluating whether a heart-failure (HF) polygenic risk score (PRS) improves risk prediction in younger patients with atrial fibrillation (AF).^1^ The authors report modest incremental discrimination and a stronger relative association of higher PRS with HF in those <60 years, an observation consistent with the notion that genetic risk exerts greater influence earlier in life.

## Key considerations

1. Ancestry and generalizability. The cohort comprised 21 167 White participants. While this reduces population stratification, it limits transferability of PRS performance to non-European populations, where allele frequencies and baseline risks differ substantially. Broader, multi-ancestry validation (or ancestry-specific calibration) is needed before clinical translation.
2. PRS handling and validation. Grouping patients into PRS tertiles—then merging the middle and highest tertiles—simplifies analysis but blunts risk gradients and obscures the extreme high-risk tail. Modelling PRS continuously (or isolating the top decile/quintile) would better capture dose–response and preserve information. Equally important, performance was assessed only within UK Biobank; external validation is essential to guard against optimism and to confirm calibration and clinical utility.
3. Subgroup power and multiplicity. The striking age interaction rests on relatively few events in the <60 subgroup, yielding wide confidence intervals and uncertainty; replication in larger young-AF datasets is warranted. In addition, multiple interactions were tested without formal adjustment, increasing the chance of false-positive subgroup signals.
4. Clinical confounding. AF phenotype (paroxysmal vs. persistent), AF burden, rate/rhythm-control strategies, catheter ablation, and cardio-protective pharmacotherapy were unavailable. Differences in AF severity, the implementation of integrated AF-CARE/ABC care, or rhythm-control uptake could partly explain higher HF incidence in high-PRS groups.^2–5^

## Future directions

Rigorously validate and, if necessary, recalibrate the HF-PRS across diverse ancestries; treat PRS as a continuous predictor variable or employ finer risk strata, reporting discrimination, calibration, and decision-analytic metrics with robust external validation; seamlessly integrate AF phenotype/burden, AF-CARE/ABC adherence, rhythm control and ablation strategies, and HF-preventive medications into comprehensive multivariable models to clearly separate genetic from modifiable risk and to rigorously test treatment–genetic interactions guiding precision therapy. Implementation for high-PRS young-AF patients must strategically pair earlier, aggressive risk-factor optimization with proactive rhythm control initiation, alongside targeted education and rigorous cost-effectiveness evaluation.

## Conclusion

Ahn *et al*. advance the case for genomic risk stratification in AF, particularly among younger patients. Addressing ancestry diversity, optimizing PRS modelling, ensuring external validation, and incorporating AF phenotype and management will strengthen generalizability and clinical usefulness. With these refinements, PRS-informed strategies could help identify truly high-risk individuals earlier and support targeted interventions to prevent HF.

## Authors’ contributions

B.D.: writing original draft, review & editing. W.L.: drafting and critical revision.
